# Large Language Model Synergy for Ensemble Learning in Medical Question Answering: Design and Evaluation Study

**DOI:** 10.2196/70080

**Published:** 2025-07-14

**Authors:** Han Yang, Mingchen Li, Huixue Zhou, Yongkang Xiao, Qian Fang, Shuang Zhou, Rui Zhang

**Affiliations:** 1 Institute for Health Informatics, University of Minnesota, Minneapolis, MN, United States; 2 Division of Computational Health Sciences, Department of Surgery, University of Minnesota, 308 Harvard Street SE, Minneapolis, MN, 55455, United States, 1 612-626-8654; 3 School of Industrial and Systems Engineering, Georgia Institute of Technology, Atlanta, GA, United States

**Keywords:** large language models, ensemble learning, medical question answering, healthcare AI, GPT-4

## Abstract

**Background:**

Large language models (LLMs) have demonstrated remarkable capabilities in natural language processing tasks, including medical question-answering (QA). However, individual LLMs often exhibit varying performance across different medical QA datasets. We benchmarked individual zero-shot LLMs (GPT-4, Llama2-13B, Vicuna-13B, MedLlama-13B, and MedAlpaca-13B) to assess their baseline performance. Within the benchmark, GPT-4 achieves the best 71% on MedMCQA (medical multiple-choice question answering dataset), Vicuna-13B achieves 89.5% on PubMedQA (a dataset for biomedical question answering), and MedAlpaca-13B achieves the best 70% among all, showing the potential for better performance across different tasks and highlighting the need for strategies that can harness their collective strengths. Ensemble learning methods, combining multiple models to improve overall accuracy and reliability, offer a promising approach to address this challenge.

**Objective:**

To develop and evaluate efficient ensemble learning approaches, we focus on improving performance across 3 medical QA datasets through our proposed two ensemble strategies.

**Methods:**

Our study uses 3 medical QA datasets: PubMedQA (1000 manually labeled and 11,269 test, with yes, no, or maybe answered for each question), MedQA-USMLE (Medical Question Answering dataset based on the United States Medical Licensing Examination; 12,724 English board-style questions; 1272 test, 5 options), and MedMCQA (182,822 training/4183 test questions, 4-option multiple choice). We introduced the LLM-Synergy framework, consisting of two ensemble methods: (1) a Boosting-based Weighted Majority Vote ensemble, refining decision-making by adaptively weighting each LLM and (2) a Cluster-based Dynamic Model Selection ensemble, dynamically selecting optimal LLMs for each query based on question-context embeddings and clustering.

**Results:**

Both ensemble methods outperformed individual LLMs across all 3 datasets. Specifically comparing the best individual LLM, the Boosting-based Majority Weighted Vote achieved accuracies of 35.84% on MedMCQA (+3.81%), 96.21% on PubMedQA (+0.64%), and 37.26% (tie) on MedQA-USMLE. The Cluster-based Dynamic Model Selection yields even higher accuracies of 38.01% (+5.98%) for MedMCQA, 96.36% (+1.09%) for PubMedQA, and 38.13% (+0.87%) for MedQA-USMLE.

**Conclusions:**

The LLM-Synergy framework, using 2 ensemble methods, represents a significant advancement in leveraging LLMs for medical QA tasks. Through effectively combining the strengths of diverse LLMs, this framework provides a flexible and efficient strategy adaptable to current and future challenges in biomedical informatics.

## Introduction

Question answering (QA) tasks in the medical domain involve a complex process of accurately interpreting and responding to health care–related queries [1]. QA tasks typically encompass two formats: open-ended and structured. In open-ended QA, respondents provide a complete sentence incorporating essential information in response to a question. In structured QA, the question is presented with several options, and the respondent selects the correct option or options by its corresponding identifier. Medical QA systems are designed to provide reliable and precise answers to questions ranging from disease symptoms and treatment options to medical research findings [2]. These systems leverage advanced technologies like natural language processing (NLP) and machine learning approaches to understand and process medical terminology and concepts, making them invaluable tools for health care professionals and patients seeking medical information. The effectiveness of these systems is crucial, as they directly impact health care decision-making and patient care [3-7].

Various models have been used for medical QA. Previously, transformer models like Bidirectional Encoder Representations from Transformers (BERT) [8] played a pivotal role in QA. For instance, He et al [9] infused disease knowledge into a basket of BERT-based models for health QA, demonstrating the viability of disease knowledge infusion in NLP models. Alzubi et al [10] developed another BERT-based model named CoBERT specifically designed for COVID-19–related QA. These BERT-based models have demonstrated solid performance on COVID-19 QA tasks but achieve substantially lower accuracy on structured, domain-specific medical QA benchmarks (eg, ≲75% vs >90% for GPT-4 on PubMedQA [a dataset for biomedical question answering]), since the expansion of medical corpora and textual resources has necessitated leveraging these large datasets more effectively. This need has been met by the emergence of large language models (LLMs) as a transformative approach to medical QA tasks. Pretrained on extensive and diverse datasets, LLMs like GPT-4 possess a deep understanding of language nuances and medical terminology, enabling them to generate highly accurate and relevant responses to medical queries [11,12]. They represent a significant milestone while dealing with NLP tasks [13], such as text generation [14,15], QA [4,7,14,16-20], Named Entity Recognition [21-25], and so on. Moreover, LLMs include large-sized models like GPT-4 [26] and Llama series (Llama-2, Llama-3, etc) [27-29], as well as some relatively small yet efficient LLMs like Vicuna [30] and Stanford Alpaca. These LLMs, characterized by their vast size, have demonstrated remarkable capabilities in understanding and generating human language across a diverse array of domains [25] as single-model approaches.

Despite these advancements, achieving satisfying performance in medical QA using off-the-shelf LLMs remains underexplored. One primary issue is the phenomenon of “hallucination,” where an LLM may generate erroneous answers due to incorrect medical knowledge or reasoning [31-33]. For example, Ji et al [31] reported that Vicuna and Alpaca-L exhibited fact inconsistency rates of 10.4% and 17.6%, respectively, in their study, underscoring the risks in high-stakes medical applications. Besides, though many LLMs are available, determining an optimal LLM with superior performance in arbitrary medical question types remains nontrivial, as the performance may vary significantly with different network architectures, training approaches, training corpora, and test question types [33]. For example, MedAlpaca [34] was fine-tuned on corpora including Wiki-doc–generated QA pairs, while MedLlama was trained on more complex clinical notes (eg, MIMIC datasets). Consequently, MedAlpaca may excel at answering short, fact-based questions such as “What is the normal range for blood pressure?,” while MedLlama may be better suited for medical questions involving nuanced patient information, such as “What is the most possible diagnosis for this patient?”

Given that different LLMs have unique characteristics and strengths, ensemble learning, which involves combining diverse models for superior performance, presents a promising approach to alleviate the above issues in medical QA [35]. First, ensemble techniques such as voting [36], boosting [37], and bagging can reduce the impact of hallucinations by filtering out erroneous responses [38]. Specifically, these methods can aggregate outputs from multiple models, effectively neutralizing inaccuracies in individual predictions. Second, ensemble techniques can harness the diverse strengths of the base models by adaptively assigning more weights to the answers from the best-suited base models, thus achieving superior performance on various question types [39,40].

However, it is challenging to effectively ensemble diverse LLMs for medical QA tasks, especially within the medical domain [41]. This integration must consider factors such as the compatibility of different models, the method of aggregating their outputs, and maintaining the interpretability of the responses [42-46]. These considerations are crucial for ensuring that the ensemble not only performs well but also aligns with the stringent requirements of medical applications. There have been only a few studies diving into Ensembling LLMs to achieve better prediction, like LLM-Blender [47], which implements PairRanker and Genfuser as an ensemble framework to generate consistently better responses for a given input. Similarly, the majority voting method proposed by Pitis et al [48] also demonstrates potential. However, these studies primarily focus on open-ended tasks and do not delve into the specifics of medical QA, nor do they include domain-specific LLMs like PMC-LlaMA2 [18] or MedAlpaca [34]. Despite substantial progress, single-model LLMs exhibit inconsistent accuracy across varied medical QA datasets due to their inherent limitations, such as domain-specific knowledge gaps [49], model biases [50], and variability in contextual understanding [51]. While general ensemble approaches exist, current methods have rarely targeted medical-specific QA tasks explicitly and have not sufficiently addressed the dynamic nature and context-specific demands of biomedical queries. Thus, there remains a critical research gap in developing robust, specialized ensemble strategies specifically tailored to enhance medical QA tasks.

To address these limitations, we introduce LLM-Synergy, a novel ensembling framework tailored for medical QA, with two well-designed meta-learning ensembling methods, providing two innovative approaches combining the strengths of various LLMs, named Boosting-based Weighted Majority Vote Ensemble [48,52,53] and Cluster-based Dynamic Model selection [53,54]. To validate the efficacy of LLM-Synergy and its ensemble methods, we conducted a case study using 3 medical QA datasets: MedMCQA (medical multiple-choice question answering dataset) [55], PubMedQA [56], and MedQA-USMLE (Medical Question Answering dataset based on the United States Medical Licensing Examination) [57].

Our contributions to this study include the following:

1. We developed innovative LLM ensemble methods, specifically the Boosting-based Weighted Majority Vote Ensemble and Cluster-based Dynamic Model Selection, which offer new approaches, with zero-shot cases, in the medical QA field.
2. We implemented the ensemble methods for LLM methods and improved the performance by 5.98%, 1.09%, and 0.87% compared to the best-performing LLM on 3 medical QA datasets, demonstrating the effectiveness of our ensemble methods. In each case, a tailored ensemble framework was created and adapted to the format of the QA dataset (single-choice or multiple-choice formats).
3. We conducted an error analysis to provide insights and directions for potential future enhancements in the field of medical QA, laying the groundwork for further improvements in this domain.

## Methods

### Overview

**Figure 1.**
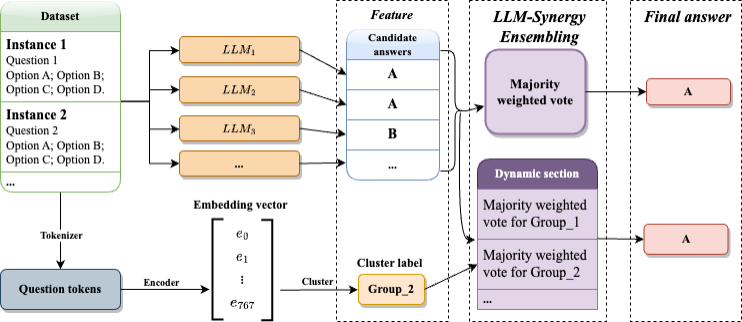
Overview of our large language model synergy framework. LLM: large language model.

The first step of our methods involves benchmarking leading individual LLMs, including GPT-3.5-turbo, GPT-4, Llama2-13B, Vicuna-13B, MedAlpaca-13B, MedLlama-13B, PMC-Llama-13B, and a random guessing baseline, to evaluate their initial performance on medical QA tasks before applying the LLM-Synergy framework. Within the benchmark, we conduct a sampled test, randomly drawing 200 QA pairs from the 3 medical QA datasets to assess the current capabilities of these LLMs in a medical context as a starting point. This benchmarking serves as a foundational analysis to understand the individual strengths and limitations of each LLM in handling medical QA tasks. Illustrated by Figure 1, which provides an overview of the whole pipeline of LLM-Synergy, following the benchmark assessment, the next phase focuses on the training process of our two proposed ensembling methods within LLM-Synergy: the Boosting-based Weighted Majority Vote Ensemble and the Cluster-based Dynamic Model Selection. The two approaches are designed to combine the unique capabilities of the selected LLMs, aiming to enhance the overall performance of medical QA systems. Moreover, the second method could be regarded as an extensive version of the first one. By implementing these methods, we seek to address the shortcomings of relying on single models and reduce the need for extensive individual model training, thereby creating a more robust and efficient solution for medical QA.

### Dataset

We used 3 medical QA datasets for our model training and test: MedMCQA [55], PubMedQA [56], and MedQA-USMLE [57].

MedMCQA, released in March 2022 by Pal et al [55], is a comprehensive multiple-choice question dataset derived from mock and past examinations of All India Institute for Medical Sciences and National Eligibility cum Entrance Test for Postgraduate (Pal et al, 2022 [58]), 2 prominent Indian medical entrance exams. It encompasses a training set with 182,822 questions and a test set comprising 4183 questions, covering more than 2400 topics. Each question in this dataset presents 4 answer choices, labeled from A to D.

PubMedQA, introduced in September 2019 by Jin et al [56], is a QA dataset curated from PubMed abstracts. It includes 1000 questions reviewed by experts and 272,500 algorithmically generated QA pairs. The dataset’s primary task is to classify research questions into yes, no, or maybe answers, akin to multiple-choice questions. It is divided into three segments: PQA-L with 1k manually labeled pairs, PQA-U with 61.2k unlabeled pairs, and PQA-A featuring 211.3k artificially generated pairs. Here, we only implement a QA process without reasoning, which does not require a corresponding explanation of how the final answer is generated, which may lead to a relatively high accuracy score.

MedQA-USMLE, launched in September 2020 by Jin et al [57], is an innovative dataset of multiple-choice questions tailored to the United States Medical Licensing Exams. This dataset encompasses questions in three languages: English, Simplified Chinese, and Traditional Chinese, with a total of 12,724, 34,251, and 14,123 questions in each respective language. Each question offers 5 choices, ranging from option A to E, sourced from professional medical board examinations. Here, we only experimented with the English QA parts. The detailed LLM prompt can be found in the Multimedia Appendices 1-3.

### LLM Benchmark on the 3 Medical QA Datasets

Ahead of implementing our ensembling framework, as a benchmark study, we evaluate the performance of various LLMs on 200 questions from each of the three QA datasets, respectively: PubMedQA, MedQA-USMLE, and MedMCQA, adhering to their specific answer formats. We evaluate our model’s performance against several robust baselines relying on the language model (LLM). Table 1 summarizes the 6 LLMs and one random guess predictor, along with the model characteristics including how many parameters within each LLM and a comprehensive description.

Table 2 reports the performance of the selected predictor in answering the 3 medical QA datasets. The benchmark graph illustrates that each predictor exhibits performance levels that exceed random guessing across various medical QA tasks, signaling the inherent capability of the LLMs to understand and process medical queries. Notably, different LLMs demonstrate particular strengths depending on the dataset; for instance, GPT-4 shows a marked proficiency in the MedMCQA tasks, while PMC-Llama -13B stands out in the PubMedQA context. These variations in model performance across tasks provide a solid foundation for the potential enhancement of accuracy through our subsequent ensemble work, suggesting that strategic combinations of these models could capitalize on their respective strengths and mitigate their individual weaknesses. For hyperparameters, we set the temperature to be 0.1 for the least randomness, and the rest, like top_p, top_k, and so on, are all set to default. The detailed LLM prompt can be found in the Multimedia Appendices 1-3.

**Table 1.** The summarization of 7 large language models running Question Answering models.

QA predictors	Model parameters	Description
GPT-4	1.76 trillion	GPT-4 is a substantial multimodal model designed to respond to questions by providing instructions fed to the GPT-4.
GPT-3.5-turbo	20 billion	Same design as GPT-4; however, GPT-3.5-turbo has fewer parameters than GPT-4.
Llama2-13B	20 billion	Llama 2-13B is part of the Llama 2 series, representing a pretrained generative model. Tuned versions of Llama 2 use supervised fine-tuning and reinforcement learning with human feedback to help generate the answers to given questions.
Vicuna-13B	13 billion	Vicuna-13, similar in size to Llama2-13b, Vicuna is noted for its robustness and adaptability across different types of language processing tasks.
MedAlpaca-13B	13 billion	MedAlpaca-13B is a substantial language model finely tuned for tasks in the medical domain. It stems from Llama (Large Language Model Meta AI) and boasts a significant parameter count of 13billion.
MedLlama-13B	13 billion	MedLlama-13B is initialized from LLaMA-13B and undergoes additional pretraining using a constructed medical corpus derived from 4.8M Pubmed Central papers and Medical Books.
PMC-Llama-13B	13 billion	PMC-Llama-13B is the further tuned version of MedLlama-13B, with the pretrain and instruction-tuning methods.
Random Guess	1	A random guess predictor simply generates a random answer by the equal probability of each option, serving as a reference to compare the large language model–cased predictor.

**Table 2.** Benchmarking the accuracy of each large language model performs on three medical Question Answering datasets.

LLMs	MedMCQA	PubMedQA	MedQA-USMLE
*GPT-4* **^a^**	*.71^a^*	0.59	0.665
GPT-3.5-Turbo	0.49	0.515	0.42
Llama2-13B	0.34	0.84	0.24
Vicuna-13B	0.315	*0*.895	0.245
MedAlpaca-13B	0.33	0.695	*0.7*
PMC-Llama-13B	0.335	0.67	0.58
Random Guess	0.23	0.5	0.165

a The italicized values indicate the highest performance among each dataset respectively.

### Part 1: Boosting-Based Weighted Majority Vote

**Figure 2.**
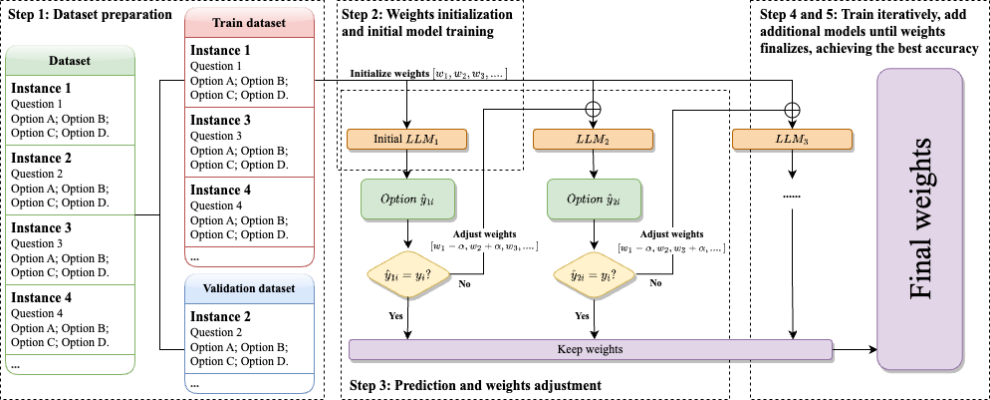
Training process of boosting-based weighted majority vote ensemble. LLM: large language model.

Figure 2 shows the training process of Boosting-based Weighted Majority Vote Ensemble.

#### Step 1: Dataset Preparation

For a given medical QA dataset, we stratified split the dataset into a training set and a validation set, with a proportion of 80% and 20%, with a fixed random seed (42 in default in our case), to preserve the same answer‐option distributions in two sets. Each QA-pair instance in the dataset consists of a question and single-choice options.

#### Step 2: Weight Initialization and Initial Model Training

We assigned initial weights to all LLMs with weights ${(w}_{1},w_{2},I)$ and initialized the starting status of ensembled LLMs with these weights. There may be different strategies for initialization, and we chose equally weighted initialization, which is the most common way of initialization [52]. For example, in our case, the equal weights of each LLM are assigned as:


$$w_{j}=\frac{1}{M},j=1,2,\ldots ,M$$

where, M denotes the number of LLMs applied.

And for each training instance i, we define an initial indicator function for prediction correctness:


$$\in_{j}\left(i\right)=\left\{ \begin{array}{l} 0,\;if\;LLM_{j}\;correctly\;predicts\;instance\;x_{i}\\ 1,\;if\;LLM_{j}\;incorrectly\;predicts\;instance\;x_{i} \end{array}\right.$$

Then the initial cumulative error, denoted as $E_{j}$ for $M_{j}$, can be computed as:


$$E_{j}=\frac{\sum_{i=1}^{N}\in_{j}\left(i\right)}{N}$$

where N is the total number of training instances.

#### Step 3: Prediction and Weight Adjustment

Next, we chose a series of LLMs, denoted as $LLM_{j}$each time, and focused on its wrong prediction: use $LLM_{j}$ to predict answers for the training set and adjust the weights based on the prediction of instance: If the prediction, $\hat{y_{1i}}$ for instance i, is incorrect, minus the weight of its corresponding LLM, $LLM_{j}$, by an adjustment parameter $\alpha_{j}$, i.e. $w_{i}\leftarrow w_{i}+\alpha_{j}$, where $\alpha_{j}$ is a factor that increases the weight, indicating that the instance needs more attention in the next round of training. The weight adjustment factor $\alpha_{j}$ for each LLM is calculated as follows:


$$\alpha_{j}=\frac{1}{2}In\left(\frac{1-E_{j}}{E_{j}}\right)$$

with this adjusting strategy, we shall be able to ensure the models performing better (lower $E_{j}$ receive larger positive adjustments, whereas poorly performing models (high $E_{j}$) obtain smaller or even negative adjustments.

#### Step 4: Iterative Training With Additional Models

For each subsequent *j −*1 LLM ($LLM_{2},LLM_{3}...$), repeat the prediction process. If a model correctly predicts the answer, we maintain the current weights. If the prediction $\hat{y}$ is incorrect, the weights were adjusted again. During the process, the implicit cost function guiding these weight updates can be interpreted as:


$$L=\sum_{j=1}^{M}\sum_{i=1}^{N}exp\left(-{\hat{y}}_{ji}f\left(x_{i}\right)\right)$$

where $f\left(x_{i}\right)$ is the weighted vote prediction of predictions:


$$f(x_{i})=\sum_{j=1}^{M}w_{j}h_{j}(x_{i})$$

where $h_{j}(x_{i})$ is the binary prediction (options chosen for our case) of $LLM_{j}$.

#### Step 5: Finalize Weights of the Model Ensemble

The iterative weight-update process continues until convergence, defined as the prediction accuracy stabilizing within a predefined small threshold (ie, accuracy change below a certain threshold) over two consecutive iterations. The training and weight adjustments across all LLMs are complete, and the final ensemble model is formed, combining the individual LLMs with the final set of adjusted weights.

### Part 2: Cluster-Based Dynamic Model Selection

**Figure 3.**
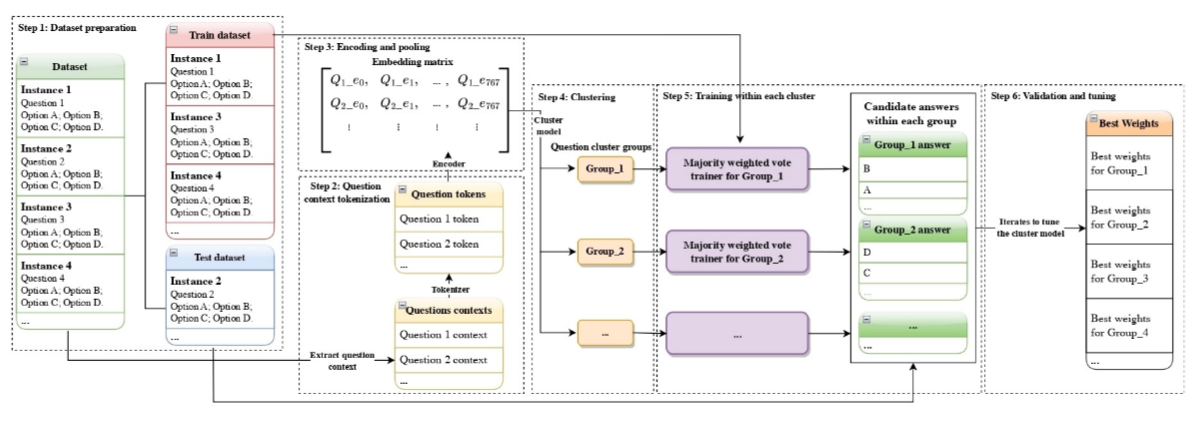
Training process of Cluster-based dynamic model selection ensemble.

Figure 3 shows the training process of our second approach: Cluster-Based Dynamic Model Selection, which serves as an extensive approach to the first one.

#### Step 1: Dataset Preparation

Same as Part 1, for a given medical QA dataset, split it into a training set and a validation set with a ratio of 80:20. Each QA-pair instance in the dataset should consist of a question paired with single-choice options.

#### Step 2: Question Context Tokenization

For each question, we first define the “question context,” which refers explicitly to the textual description and details provided within each question instance (excluding the candidate answer options). We then tokenize each question context using a common tokenizer model (in our case, Clinical-BERT [59], which was pretrained on large-scale clinical notes and outperformed general biomedical models, was chosen since it matches our Medical QA situation very well), transforming the textual information into sequences of numerical tokens.

#### Step 3: Encoding and Pooling

Next, we encode these numerical token sequences using Clinical-BERT (same as step 2) to generate high-dimensional semantic representations. Specifically, we use mean-pooling over Clinical-BERT’s final hidden states, obtaining a 768-dimension embedding vector for each question. As a result, all question embeddings collectively form an embedding matrix of size N×768, where N is the total number of questions.

#### Step 4: Clustering

Apply a clustering model, KMeans (K-Means Clustering Algorithm) [60] in our experiment specifically, directly to the embedding matrix. This step assigns each question a cluster label, effectively reducing the dimensionality from 768 of an individual question vector to a single cluster group label being 1,2, 3, … K. Thereafter, each question’s embedding vector is thus assigned to one of K clusters (labels: 1, 2, 3, …, K), effectively grouping questions that have similar semantic characteristics. In our experiment, the optimal K was chosen based on the highest mean silhouette coefficient [61] and confirmation by the elbow method [62] on within-cluster sum of squares, to ensure both cluster compactness and stability.

#### Step 5: Training Within Clusters

For each cluster group identified previously, we implement the Boosting-based Majority Weighted Vote ensemble method (outlined earlier in Part 1). Then for each cluster, the ensemble weights for the LLMs are adjusted specifically to maximize predictive accuracy on questions belonging to that cluster. In other words, each cluster obtains its own optimized combination of LLM weights, tailored precisely to the context characteristics of the cluster.

#### Step 6: Validation and Hyperparameter Tuning

Finally, we evaluate the ensemble’s predictive performance on the validation set by repeating Steps 2 through 5, and iteratively adjust and tune the hyperparameters of the clustering algorithm (K in KMeans in our case), and any other hyperparameters (such as KMeans initialization settings) to achieve the best overall accuracy on the validation set. This ensures the clustering algorithm effectively captures meaningful patterns in question contexts, thereby optimizing our dynamic selection ensemble’s final accuracy.

### Evaluation

In the evaluation phase of our study, same as others [18,47,55,57], accuracy was used as the primary metric for assessment, given its congruence with the micro *F*_1_-score in our specific experimental context of single-choice or multiple-choice QA tasks. Given the nature of multiple-choice QA datasets, metrics such as Recall and Precision are deemed inappropriate as they are sensitive to changes in the option numbering. For instance, adjusting the order of option labels may result in altering these metric values when the number of options exceeds 2. Based on these considerations, accuracy emerges as a more suitable evaluation metric in our case, providing a robust measure of overall correctness without being affected by variations in option numbering, which ensures a consistent and meaningful assessment of model performance in the specific context of our study.

The test set is distinguished from the training set used for model development and the validation set for hyperparameter optimization that we used within the ensemble training process. This distinct separation ensures an unbiased evaluation of the model’s true predictive capabilities. The test sets are derived from the subsets of the MedMCQA, PubMedQA, and MedQA-USMLE datasets, respectively. The MedMCQA test dataset consists of 4183 QA pairs. The PubMedQA test dataset is even more extensive with 11,269 QA pairs, whereas the MedQA-USMLE dataset contains 1272 QA pairs.

### Ethical Considerations

Institutional review board exemption was granted because the study made use of publicly available, deidentified datasets and did not involve human subjects research as defined by 45 CFR 46.

## Results

In this study, the performance of various LLMs was evaluated against ensemble methods across 3 distinct medical QA datasets, MedMCQA, PubMedQA, and MedQA-USMLE. The performance metric, assumed to be an accuracy score, highlights the differential capabilities of each LLM and our two established predictors of ensemble methods. The detailed result can be seen in Table 3.

For individual models, Llama2-13B demonstrated substantial proficiency in the PubMedQA dataset with an accuracy of 93.09%, indicating a strong alignment with the dataset’s characteristics. Conversely, its performance on the MedQA-USMLE dataset was considerably lower at 24.61%, suggesting a potential misalignment with this dataset’s attributes or a limitation in handling its complexity. MedLlama-13B showed a similar trend, albeit with marginally lower accuracy figures across the board, peaking at 86.11% for the PubMedQA dataset.

MedAlpaca-13B yielded a divergent performance profile, exhibiting a relatively lower accuracy of 27.97% on PubMedQA, while achieving the highest accuracy among individual LLMs on the MedQA-USMLE dataset at 37.26%. This suggests that MedAlpaca-13B may possess particular strengths in processing the content typified within the MedQA-USMLE exam questions. Vicuna-13B, on the other hand, had the lowest accuracy scores for all datasets, which could indicate a general difficulty with the medical QA task as presented in these datasets.

**Figure 4.**
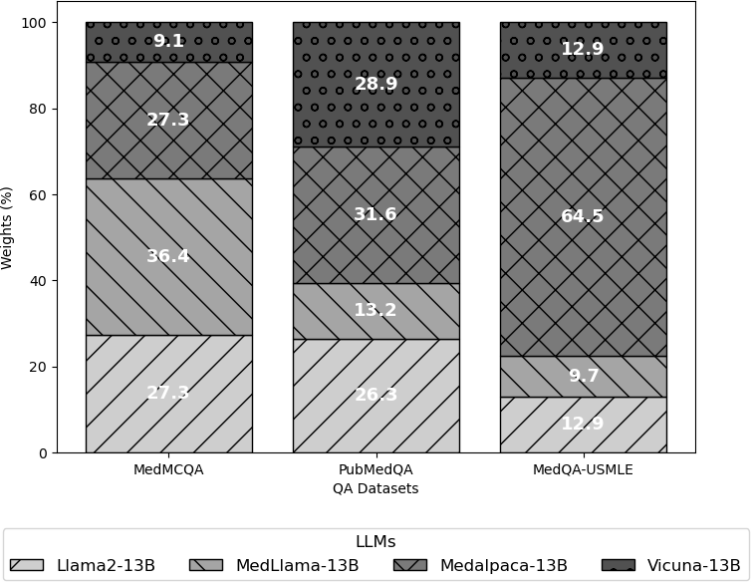
Weights of the component large language models under each Question Answering dataset.

The ensemble approaches, notably the Boosting-based Weighted Majority Vote ensemble and the Cluster-based Dynamic Selection ensemble, were developed to leverage the collective strengths of the individual LLMs. The Boosting-based Weighted Majority Vote Ensemble surpassed the individual model performances on the MedMCQA and MedQA-USMLE datasets and achieved a notable accuracy of 96.21% on PubMedQA, and the detailed weights of component LLMs are disclosed in Figure 4. This enhancement suggests that a static weighted combination of model outputs can capitalize on the diverse expertise of each LLM to improve overall performance. Whereas the Cluster-based Dynamic Selection Ensemble, which introduces a context-aware model selection strategy, further improved upon the Boosting-based Weighted Majority Vote ensemble’s performance, achieving the highest accuracy across all datasets: 38.01% on MedMCQA, 96.36% on PubMedQA, and 38.13% on MedQA-USMLE. The specific range of improvement varies from the variation of each LLM. The final cluster numbers are 9 in MedMCQA, 12 in PubMedQA, and 5 in MedQA-USMLE.

**Table 3.** Test set performance of individual large language model and our ensemble approach.

LLMs	MedMCQA	PubMedQA	MedQA-USMLE
Llama2-13B	32.03	93.09	24.61
MedLlama-13B	31.03	86.11	23.58
MedAlpaca-13B	27.97	95.27	37.26
Vicuna-13B	26.13	93.15	24.14
Boosting-based Weighted Majority Vote	35.84	96.21	37.26
Cluster-based Dynamic Model Selection	*38.01^a^*	*96.36*	*38.13*

a The italicized values indicate the highest performance among each dataset respectively.

## Discussion

### Principal Findings

In this study, we demonstrated that both methods of our proposed LLM-Synergy ensemble framework improved prediction accuracy across three medical QA datasets—MedMCQA, PubMedQA, and MedQA-USMLE—compared to individual LLMs. On MedMCQA, where base models’ accuracies ranged narrowly from 26.13% to 32.03%, —Cluster-based Dynamic Model Selection yielded the largest gain (+5.98%) by clustering semantically similar questions and tailoring weights to each cluster’s specific context. On PubMedQA, with uniformly high single-model performance (min 86.11%), Boosting-based Weighted Majority Vote already delivered a modest improvement (+0.94%) through global weight adjustments, and Cluster-based Dynamic Model Selection added a further slight boost (+1.15%) by fine-tuning weights per question group. Finally, MedQA-USMLE presented a case where one model (MedAlpaca-13B) dominated; here, Cluster-based Dynamic Model Selection’s context-sensitive reweighting prevented over-reliance on that single model and achieved a +0.87%gain over static Boosting-based Weighted Majority Vote.

The effectiveness of this design can be attributed to two key factors: First, the Boosting-based Weighted Majority Vote addresses systematic biases by amplifying the influence of models that consistently perform well across all questions. Second, the Cluster-based Dynamic Model Selection further adapts to question heterogeneity by dynamically identifying the optimal combination of models for each input. Together, these tailored strategies explain the superior performance of LLM-Synergy across varied medical QA formats. Moreover, our analysis reveals that the Cluster-based Dynamic model outperforms the Boosting-based Weighted Majority Vote on the three QA datasets. On MedMCQA, it achieves 38.01% versus 35.84% (+2.17%), on PubMedQA 96.36% versus 96.21% (+0.15%), and on MedQA-USMLE 38.13% versus 37.26% (+0.87%). This improvement likely arises from the Cluster-based Dynamic Model Selection’s ability to assign model weights tailored to semantically coherent question clusters, enabling each model to excel in areas where it performs best, rather than relying on a uniform global weighting approach.

**Figure 5.**
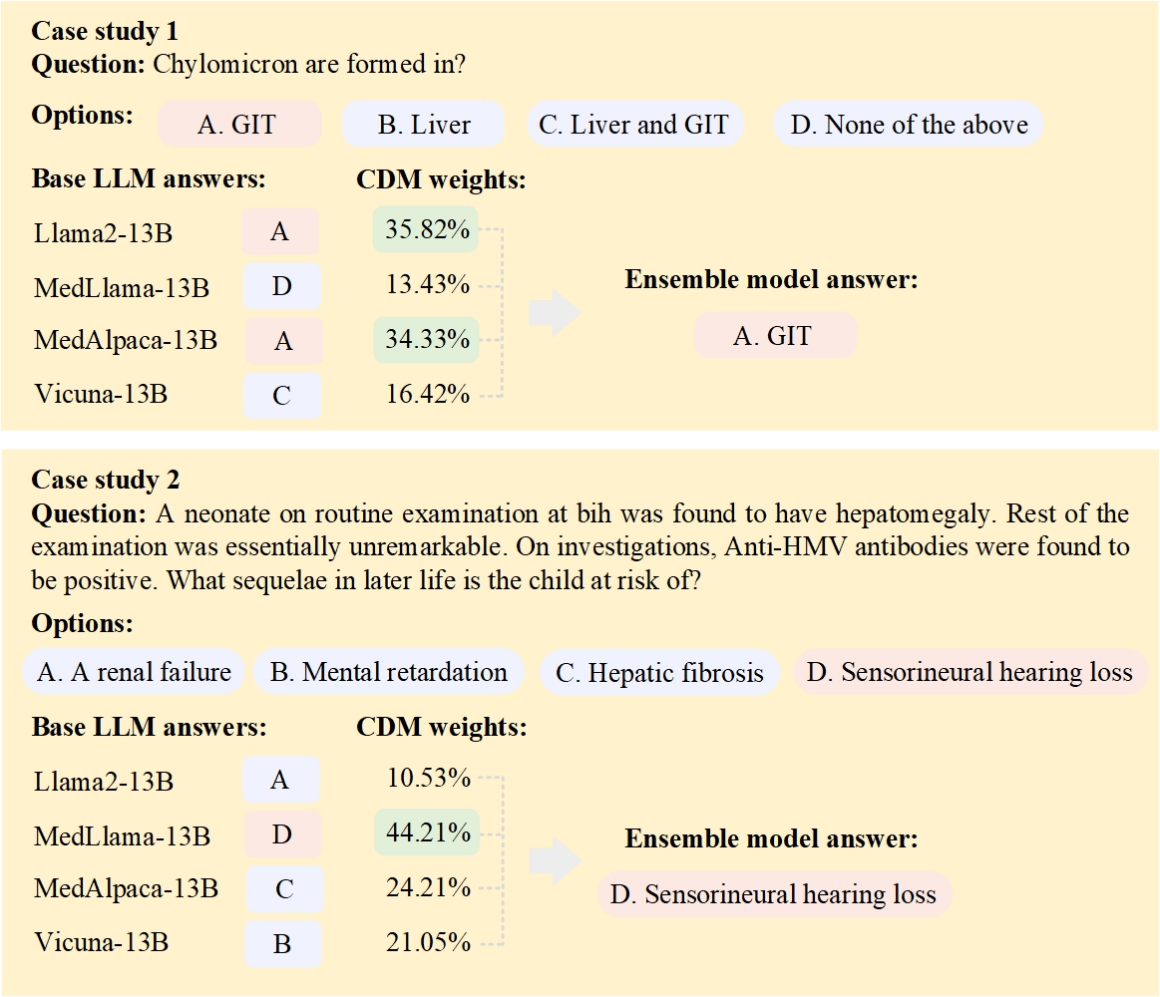
Case studies of the Cluster-based dynamic model selection on the MedMCQA dataset. The red color denotes the correct answer, and the green color highlights the base LLMs receiving large weights. The results demonstrated that the proposed ensemble model assigned larger weights to the most suitable LLMs within each question cluster, thus enhancing the likelihood of selecting the correct answer. GIT: gastrointestinal tract.

To further illustrate how the Cluster-based Dynamic Model Selection adapts model contributions to specific contexts, we visualized weight distributions for several clusters from the MedMCQA dataset. These visualizations demonstrate that assigning greater weights to the most competent LLMs within each question cluster enhances the likelihood of selecting the correct answer, thereby validating the model’s superior performance (see Figure 5). For instance, in the fact-based question (ie, the first case in Figure 5), we observed that MedAlpaca and Llama were assigned larger weight values than MedLlama (35.82% and 34.33% versus 13.43%), while in the question involving complex patient information and clinical decision-making (ie, the second case in Figure 5), MedLlama instead obtained larger weights (44.21%) than the others. This discrepancy arises from the distinct training corpora of the base models, which renders these LLMs to have different strengths. Specifically, MedAlpaca and Llama were fine-tuned on corpora including Wiki-doc–generated QA pairs and may excel at answering short, fact-based questions, while MedLlama was trained on more complex clinical notes (eg, MIMIC datasets) and may be better suited for medical questions with nuanced patient information and clinical decision-making.

### Limitations

LLM-Synergy’s effectiveness depends critically on the quality and diversity of its constituent LLMs: if these models share similar biases or blind spots, the ensemble may offer little improvement over any single model. The weighted-majority vote approach, while efficient, uses fixed weights and cannot adapt to question-specific nuances, leading to potential underperformance when individual model strengths vary by context. The cluster-based dynamic selection method addresses this by learning cluster-specific weights, but its success relies on the clustering algorithm’s ability to capture relevant question features and on having a sufficiently large, representative validation set for tuning.

Despite avoiding expensive fine-tuning, both methods incur nontrivial computational overhead when integrating multiple LLM inferences and Clinical-BERT embeddings, which may limit real-time deployment in resource-constrained environments. Additionally, both approaches assume that model performance on the validation set reliably predicts test-set behavior; substantial dataset shifts could thus degrade ensemble effectiveness. Finally, the scarcity of direct comparisons with alternative ensemble strategies or traditional fine-tuning on these specific medical QA datasets highlights the need for future work to benchmark and further optimize ensemble configurations.

### Computational Cost

Both our ensemble methods operate entirely at inference time—no fine-tuning or retraining of base LLMs is required—resulting in substantially lower overall compute than full model training. We approximate the computational complexity for the training phase as $O(N\times M\times C_{LLM})$ for method 1, Boosting-based Weighted Majority Vote Ensemble inference, where M is the number of individual LLM, N is the number of training instances (questions to answer), $C_{LLM}$ is the inference cost for each individual LLM. Whereas for method 2, Cluster-based Dynamic Model Selection, inference complexity approximately scales linearly with the number of ensemble models used $O(N\times (M\times C_{LLM}+C_{BERT}+K\times I\times d))$, where K is the chosen number of clusters, I is the number of iterations (typically small, eg, ~20‐50), and d is the embedding dimension (768 in our case).

In practice, using our experiments as an example, inference for the ensemble method takes roughly 6‐12 seconds per question on a single A100 GPU, compared to 1‐2 seconds for an individual model alone. Although multi-second latency may exceed real-time requirements, it remains modest relative to the cost of full model fine-tuning and is easily amortized in batch workflows. Moreover, latency can be reduced via parallelization across GPUs, model distillation into a compact student network, adaptive inference (invoking the full ensemble only when needed), and caching of frequent queries.

### Conclusions

The LLM-Synergy framework, with its Boosting-based Weighted Majority Vote and Cluster-based Dynamic Model Selection methods, represents a significant advancement in leveraging LLMs for medical QA tasks and provides an innovative way of efficiently using the development with LLM technologies, customizing for both existing and potentially future challenge tasks in biomedical and health informatics research. Its ability to amalgamate the strengths of multiple models has demonstrated superior accuracy and robustness over individual LLMs. While the framework showcases scalability, flexibility, and adaptability across domains, it also presents opportunities for future enhancements, including increased model diversity, dynamic weighting, and broader domain applications. As such, LLM-Synergy not only addresses current challenges in natural language processing but also sets the stage for continued innovation in artificial intelligence–driven problem-solving.

## Supplementary material

10.2196/70080 Multimedia Appendix 1 Prompt example for MedMCQA dataset, same for benchmarking and modeling.

10.2196/70080 Multimedia Appendix 2 Prompt example for PubMedQA dataset, same for benchmarking and modeling.

10.2196/70080 Multimedia Appendix 3 Prompt example for MedQA-USMLE dataset, English language, same for benchmarking and modeling.

## ABBREVIATIONS

BERT: Bidirectional Encoder Representations from Transformers
KMeans: K-Means Clustering Algorithm
LLM: large language model
MedMCQA: medical multiple-choice question answering dataset
MedQA-USMLE: Medical Question Answering dataset based on the United States Medical Licensing Examination
NLP: natural language processing
PubMedQA: a dataset for biomedical question answering
QA: question answering
