# Supplementary figures and images for: Large Language Model Synergy for Ensemble Learning in Medical Question Answering: Design and Evaluation Study
Source: J Med Internet Res. 2025 Jul 14;27:e70080. doi: 10.2196/70080 (PMC12337233; doi:10.2196/70080)

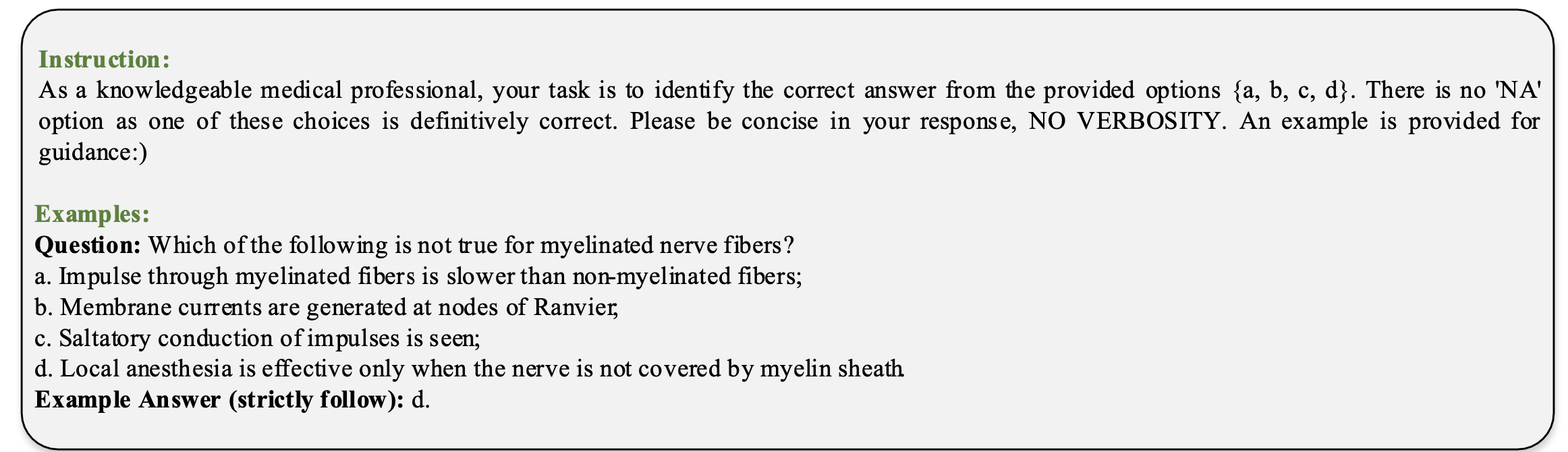

Supplement: Multimedia Appendix 1 [file jmir-v27-e70080-s001.png]

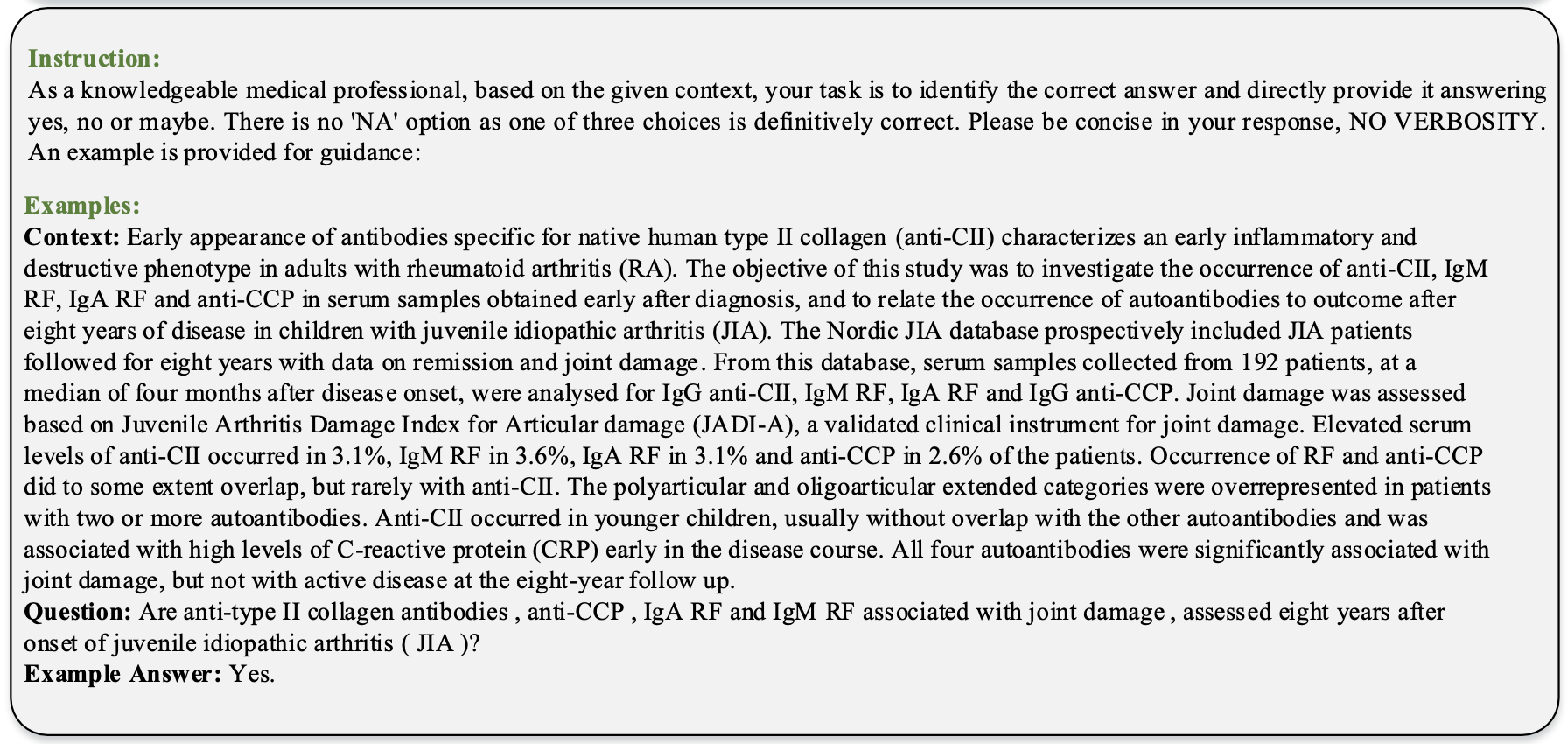

Supplement: Multimedia Appendix 2 [file jmir-v27-e70080-s002.png]

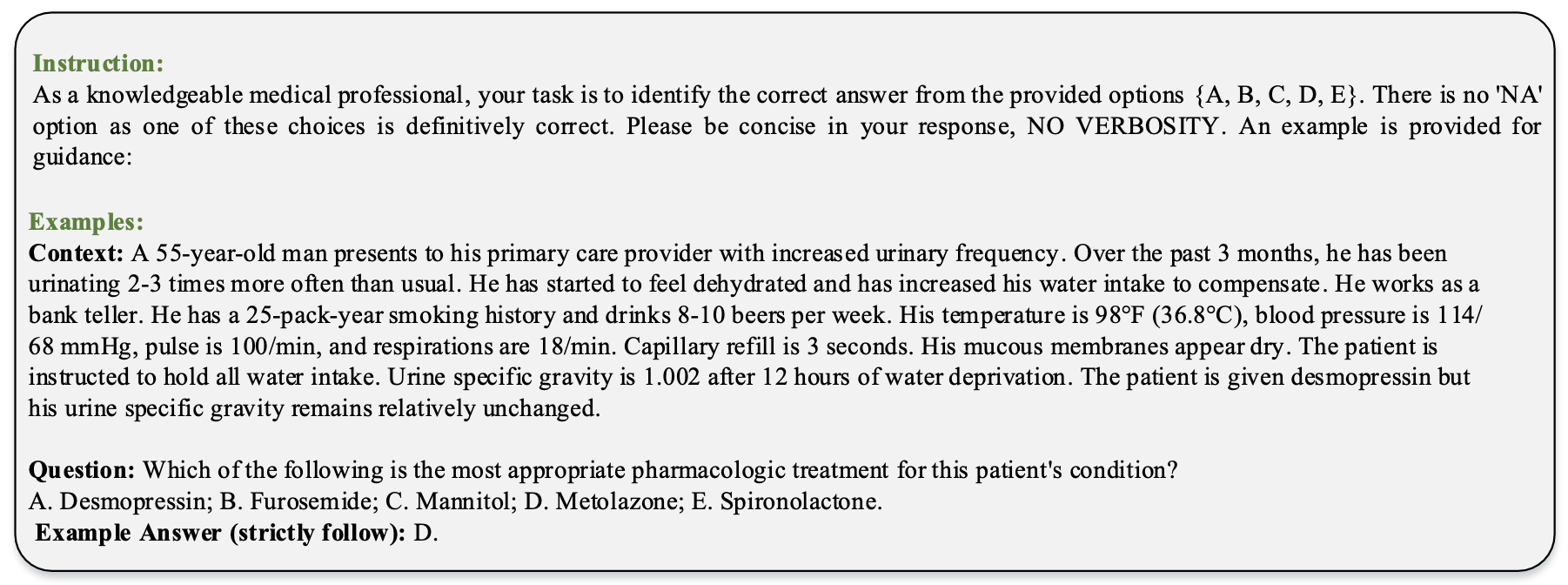

Supplement: Multimedia Appendix 3 [file jmir-v27-e70080-s003.png]
